# Evaluation of changes to work patterns in multidisciplinary cancer team meetings due to the COVID‐19 pandemic: A national mixed‐method survey study

**DOI:** 10.1002/cam4.5608

**Published:** 2023-01-17

**Authors:** Tayana Soukup, David Winters, Kia‐Chong Chua, Philip Rowland, Jacqueline Moneke, Ted A. Skolarus, Rasiah Bharathan, Leanne Harling, Anish Bali, Viren Asher, Tasha Gandamihardja, Nick Sevdalis, James S. A. Green, Benjamin W. Lamb

**Affiliations:** ^1^ Institute of Psychiatry, Psychology, and Neuroscience, Health Service and Population Research Department King's College London London UK; ^2^ Department of Urology Barts Health NHS Trust London UK; ^3^ Department of Urology Cambridge University Hospital NHS Trust London UK; ^4^ Dow Division of Health Services Research, Department of Urology University of Michigan, Center for Clinical Management Research, Veterans Affairs Ann Arbor Healthcare System Ann Arbor Michigan USA; ^5^ University Hospitals of Leicester NHS Trust Leicester UK; ^6^ Department of Surgery and Cancer Imperial College London London UK; ^7^ School of Cancer and Pharmaceutical Science Kings College London London UK; ^8^ Gynaecology Cancer Centre University Hospitals of Derby & Burton Derby UK; ^9^ Chelmsford Breast Unit Broomfield Hospital Chelmsford UK; ^10^ Bart’s Cancer Institute Queen Mary University of London London UK; ^11^ Department of Urology University London College Hospitals London UK

**Keywords:** cancer, cancer multidisciplinary team meetings, decision‐making, multidisciplinary teams

## Abstract

**Background:**

It is not well understood the overall changes that multidisciplinary teams (MDTs) have had to make in response to the COVID‐19 pandemic, nor the impact that such changes, in addition to the other challenges faced by MDTs, have had on decision‐making, communication, or participation in the context of MDT meetings specifically.

**Methods:**

This was a mixed method, prospective cross‐sectional survey study taking place in the United Kingdom between September 2020 and August 2021.

**Results:**

The participants were 423 MDT members. Qualitative findings revealed hybrid working and possibility of virtual attendance as the change introduced because of COVID‐19 that MDTs would like to maintain. However, IT‐related issues, slower meetings, longer lists and delays were identified as common with improving of the IT infrastructure necessary going forward. In contrast, virtual meetings and increased attendance/availability of clinicians were highlighted as the positive outcomes resulting from the change. Quantitative findings showed significant improvement from before COVID‐19 for *MDT meeting organisation and logistics* (*M* = 45, SD = 20) compared to the *access* (*M* = 50, SD = 12, *t*(390) = 5.028, *p* = 0.001), *case discussions* (*M* = 50, SD = 14, *t*(373) = −5.104, *p* = 0.001), and *patient representation* (*M* = 50, SD = 12, *t*(382) = −4.537, *p* = 0.001) at MDT meetings.

**Discussion:**

Our study explored the perception of change since COVID‐19 among cancer MDTs using mixed methods. While hybrid working was preferred, challenges exist. Significant improvements in the meeting organisation and logistics were reported. Although we found no significant perceived worsening across the four domains investigated, there was an indication in this direction for the case discussions warranting further ‘live’ assessments of MDT meetings.

## Graphical abstract

Multidisciplinary teams (MDTs) are a gold standard in cancer care. Qualitative findings revealed hybrid working and possibility of virtual attendance as the change introduced because of COVID‐19 that MDTs would like to maintain. IT‐related issues, slower meetings, longer lists and delays were reported as common with further improvement of the IT infrastructure critical.

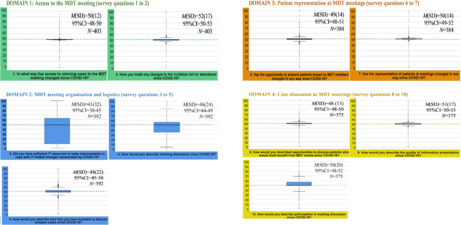

## 1 BACKGROUND

A multidisciplinary approach is accepted as the gold‐standard means of addressing the complex needs of patients with cancer.^1^, ^2^, ^3^, ^4^, ^5^ In the UK, such care planning is routinely (and mandatorily) carried out by a multidisciplinary team (MDT), generally consisting of histopathologists, radiologists, surgeons, cancer nurse specialists (CNSs) and oncologists, in typically weekly or fortnightly meetings for high‐volume cancers (or, ‘tumour boards’ in some settings). In these meetings, patients' medical history and test results are reviewed, and treatment options formulated—many large centres in the UK undergo this process in a single meeting for several hours at a time, until all patients put forward for MDT review have been discussed.^1^, ^2^, ^3^, ^4^, ^5^, ^6^ Our study will zoom in on understanding the changes, as a result of the COVID‐19 pandemic, to the MDT meetings specifically, as one particular segment of the care pathway.

The COVID‐19 pandemic has had a profound effect on all aspects of life for many people globally. Healthcare has been particularly affected, both by the increased strain on resources, and by measures imposed to limit the spread of the virus among patients and healthcare professionals.^7^, ^8^ One such measure, widely adopted globally by many industries, including healthcare, was the cessation of face‐to‐face meetings where they could be contacted remotely.^9^, ^10^ These changes were facilitated by the increasing uptake of remote videoconferencing platforms, which have been used to allow meetings to go ahead with remote interaction.^11^ MDT meetings are an example of face‐to‐face meetings that were changed to entirely remote, or hybrid with a core of personnel hosting the meeting in person, with other members logging in remotely.^12^ Interestingly, many MDTs were already using remote meetings to a degree, in order to discuss cases over distance, or to allow participation of members who were unable to travel.^13^ In keeping with many aspects of the changes that were introduced to cope with COVID‐19, changes to MDT meetings were adopted locally, often over a short time period and with little opportunity for planning or piloting of different strategies due to pandemic urgency.^12^

The pandemic aside, MDTs have been under increasing pressure from the changing economic/political landscape surrounding healthcare,^14^, ^15^ rising cancer incidence,^14^, ^15^, ^16^ severe staff shortages,^17^ steadily increasing workload,^18^ external circumstances (e.g. time‐workload pressure, number of cases for discussion, complexity of cases), team factors (e.g. team size, gender composition, disciplinary diversity),^19^, ^20^, ^21^, ^22^, ^23^ and the logistical challenges (i.e. admin errors and problem issues, attendance and equipment issues).^22^, ^23^, ^24^ in many countries, and for many cancer services, the pandemic exacerbated such pre‐existing pressures.

### 1.1 Study aims and objectives

It is not well understood the overall changes that MDTs have had to make in response to the COVID‐19 pandemic, nor the impact that such changes, in addition to the other challenges faced by MDTs, have had on decision‐making, communication, or participation in the context of their MDT meetings specifically. Furthermore, with the pandemic in relative remission, it is time cancer services established which changes brought about because of the COVID‐19 pandemic will or should be retained as beneficial adaptations in the future (post‐pandemic), and where services need to return to a pre‐pandemic status quo.

This study starts to address these questions in the context of MDT meetings specifically. The aim of the study was to explore qualitatively (objective 1) and quantitatively (objective 2) the views of MDT members on the impact of COVID‐19 on their MDT meetings, while eliciting the responses on any related changes that emerged concerning (1) access to MDT meetings, (2) meeting organisation and logistics, (3) patient representation at the meetings, and (4) quality of case reviews at the meetings.

## 2 METHODS

### 2.1 Study design

This was a prospective cross‐sectional survey study.

### 2.2 Study setting

The study took place in the United Kingdom between September 2020 and August 2021.

### 2.3 Participants

Survey participants were recruited using a snowball sampling method, using established networks with participants encouraged to share the invitation to participate through the NHS England and NHS Improvement's communication channels such as Cancer Alliances, specialty organisations, and within institutions.^25^ The invitation was aimed at MDT clinical leads, and MDT coordinators. These members were chosen as both have roles with an overview of how their team had adapted to COVID‐19, and what technology/infrastructure had been used. They were sent an introductory email (See File S1) with the web address of the survey and were encouraged to circulate the details to other MDT members. The study was registered as service evaluation or audit in each participating site. Consent was implied upon return of the survey, and response to the survey was voluntary.

### 2.4 Survey design

Our survey consisted of two complementary parts (See File S2). The first part was qualitative, eliciting free‐text responses to four questions focused on the impact of COVID‐19 on the MDT meetings specifically, including favourable changes to MDT meeting to be maintained going forward, and changes that highlight more pressing issues limiting MDT working (in the context of MDT meetings). The second part of our survey was quantitative, with 10 questions across four domains including: access to the MDT meeting (two questions), MDT meeting organisation and logistics (three questions), patient representation at MDT meetings (two questions), and case discussions at MDT meetings (three questions). Responses to the survey questions were provided on a sliding scale marker ranging from 0 to 100. Scores below 50 represented improvements from before COVID‐19 in the four survey domains. Scores above 50 represented deteriorations from before COVID‐19. A score of 50, that is, mid‐point on the sliding scale marker, represented no change from before COVID‐19. Further description of the individual variables and their scales can be found in the Additional File 2. This mixed‐methods approach increases the richness and relevance of our findings regarding MDT workings and highlights improvement opportunities.

### 2.5 Qualitative and quantitative analyses

To address objective 1, we used NVivo 12 to analyse qualitative responses inductively and deductively using a thematic approach. We used a systematic classification process of coding and identifying themes/patterns in the data with the main themes and sub‐themes formed/shaped inductively by the data.^26^, ^27^ Two assessors analysed the data and reviewed the themes (TS and BWL).

To address objective 2, we used SPSS IBM SPSS Statistics 24 where we examined group differences in survey responses by comparing descriptive statistics using boxplots, and by inferential statistics using *t*‐test. The nature of sampling (snowball) precluded calculation of a formal response rate, as the denominator (i.e. the number of cancer professionals who received the invite to participate) remained unknown throughout the study.

## 3 RESULTS

### 3.1 Participant characteristics

The participants were MDT members (*N* = 423) from England (*n* = 401), Scotland (*n* = 16), Wales (*n* = 4), and Northern Ireland (*n* = 2). The members represented different professional groups: Allied Health Professionals (AHP; *n* = 10), Cancer Nurse Specialists (CNS; *n* = 45), Coordinating, Administrative and Management (MDT‐CAM; *n* = 105), Oncologists (*n* = 67), Pathologist (*n* = 7), Physician (*n* = 36), Radiologist (*n* = 12), and Surgeons (*n* = 135). The average years of work experience in these roles was 7 with a minimum of 1 and maximum of 10 years (*n* = 82 of 1‐2 years; *n* = 118 of 3‐7 years; *n* = 202 of >8 years). A Kruskal–Wallis test revealed no statistically significant differences in the survey responses depending on the seniority levels (*p* > 0.05 on all survey questions). The participants covered 8 different cancers: Breast (*n* = 45), Colorectal (*n* = 25), Gynaecological (*n* = 103), Head and Neck (*n* = 23), Lung (*n* = 26), Skin (*n* = 22), Upper GI (*n* = 21), Urology (*n* = 102) and Other (*n* = 39). There were also no statistically significant differences in the responses between different cancer specialties (*p* > 0.05 on all survey questions). Majority of participants were from more specialist MDTs (SMDTs) hosted largely at University or Teaching Hospitals (*n* = 291), and somewhat smaller number was from local‐MDTs (LMDTs) hosted largely at District Hospitals (*n* = 129); there were no statistical differences between the two (*p* > 0.05 on all survey questions).

### 3.2 Objective 1: Thematic analysis of free‐text survey responses

The main themes for each question are presented in Table 1 alongside their frequency, example quotes, and their representation across the participating professional groups. We found hybrid working and possibility of virtual attendance was the most frequent theme in response to *what changes to MDT working will you maintain*. IT‐related issues and slower meetings, longer lists and delays were the most common theme in response to the question on whether changes have caused any specific problems. Virtual meetings and increased attendance/availability of clinicians was the most common response to the question concerning *changes that provided solutions*. Improving IT infrastructure, its use and access were cited as the most *common change that now seems more pressing than before*.

**TABLE 1:** Results from thematic analysis of the four free text response questions with the dimensions, data examples, frequencies and professional role representations (items are presented in order of their frequency of appearance in participants' responses)

Discourse and dimension	Data example (participants' written quotes)	Frequency (%)^a^ U = *n*; D = *n* ^b^	Professional roles	
1. What changes to MDT working will you maintain?		304/423 (72%) U = 212; D = 92		
Hybrid working and improved attendance	Hybrid meetings allowing for those not on site to attend. Option of virtual access. (Physician) Keep virtual as more attend. (CNS) Better attendance now more colleagues can attend virtually. (Surgeon)	258/304 (85%) U = 177; D = 81	CNS, MDT‐CAM, Path, Onc, Phy, Surg, Path, AHP	
b None	We have not noticed any significant changes in our local MDT. (Surgeon)	11/304 (4%) U = 5; D = 6	CNS, MDT‐CAM, Surg, Phy, Onc	
c Streamlining of cases	Streamlining of low risk cases. (Surgeon) Clinical prioritisation. (MDT‐CAM)	13/304 (4%) U = 10, D = 3	CNS, MDT‐CAM, Phy, Surg, Onc	
d No printed paperwork	No printed paperwork. (MDT‐CAM)	12/304 (4%) U = 10, D = 2	MDT‐CAM, Surg	
e Hybrid working highlighted importance of face‐to‐face contact	Continue as present. Also offer the option to local teams to return to meeting in a room, as this does engender better inter‐personal team relationships and communications. (Surgeon)	6/304 (2%) U = 6; D = 0	Surg, Phy, CNS, Onc	
f Adequate preparation time	Improved preparation. (Surgeon)	4/304 (1%) U = 4; D = 0	Phy, Surg	
2. Have changes caused any specific problems?		288/423 (68%) U = 204; D = 84		
IT‐related issues and slower meetings, longer lists and delays	Remote access often falls over. (Oncologist) There can be sound problems/technical problems during the MDT which increases the time needed to discuss patients. IT slowed meeting down. (Radiologist)	125/288 (43%) U = 83; D = 42	Onc, Rad, Surg, MDT‐CAM, CNS, Phy, Path, Rad, AHP	
b None	Not really. (MDT‐CAM)	92/288 (32%) U = 66; D = 26	MDT‐CAM, Path, Rad, Surg, Onc, Phy, CNS	
c Virtual meetings and problems around communication and case discussion	Less face to face affects communication. (AHP) Virtual meetings make communication more difficult. (Surgeon)	34/288 (12%) U = 26; D = 8	AHP, CNS, MDT‐CAM, Surg, Phy, Onc,	
d Virtual meetings and issues around quoracy	Occasional quoracy issues where people do not realise they are the last person from their specialty on the meeting and dial off, for example, last two surgeons both leave simultaneously. (Physician)	14/288 (5%) U = 9; D = 5	CNS, Surg, Phy, Surg, MDT‐CAM, Onc	
e Quality of patient related information	Sometimes due to people working off site patients being referred to the MDT do not always have completed information and when trying to track down the information you are often told no one is onsite and you will have to wait till they are on site. (MDT‐CAM)	6/288 (2%) U = 3; D = 3	Surg, Onc, MDT‐CAM	
f Issues around meeting room space	Lack of space for staff. (Surgeon) MDT room had to be moved to a smaller room, which is harder for social distancing. Dispersion of group across 2–3 rooms. (MDT‐CAM)	5/288 (2%) U = 5; D = 0	AHP, MDT‐CAM, Surg, CNS	
g Adjustment period to virtual/hybrid working	Took some time to settle into using Teams although is fine now. (MDT‐CAM)	5/288 (2%) U = 5; D = 0	MDT‐CAM, Onc, Path	
h Increased caseload and complexity of patients	Higher volume of emergency presentations with advanced cancer has meant cases are more complex and there is more time pressure. (Oncologist)	3/288 (1%) U = 0; D = 0	Surg, Onc, CNS	
i Increased caseload and stress/pressures on MDT members	Backlog of patients has tested capacity. (Radiologist) Increased stress to MDT members. (Physician)	3/288 (1%) U = 3; D = 0	Phy, Surg, Rad	
j Reduced teaching opportunities	Reduced teaching opportunities for students and junior doctors. (Oncologist)	1/288 (0.3%) U = 1; D = 0	Onc	
3. What changes provided solutions?		252/423 (60%) U = 170; D = 82		
Virtual meetings and increased attendance/availability of clinicians	Improved availability of those needed for MDT. (Surgeon) Yes, it is more accessible for members to join the meeting and ask questions, present last minute complex cases and so on. (MDT‐CAM) Pathology input improved as able to participate virtually. (Surgeon)	103/252 (41%) U = 74; D = 29	MDT‐CAM, Surg, CNS, Phy, Onc, Rad,	
b None	No, I do not think so. (AHP)	78/252 (31%) U = 60; D = 18	MDT‐CAM, AHP, CNS, Onc, Rad, Surg, Phy,	
c Improved IT infrastructure, its access and use	IT much better than prior to COVID. Development of e‐referral to Breast MDT. (Surgeon) Easier for everyone to see scans / histology. Swifter and better functioning IT. (Oncologist)	30/252 (12%) D = 14; U = 16	Surg, Onc, Phy, AHP, CNS, MDT‐CAM, Path	
d Virtual meetings and a more streamlined approach (to discussion, communication and case selection)	Yes, a more streamlined approach with less people all talking at once due to the remote nature of the MDT. (MDT‐CAM) We have streamlined the cancer clinic to accommodate those patients who need face‐to‐face appointments. (MDT‐CAM) Microsoft team allows discussions to be more streamlined and not talking over each other. (CNS)	19/252 (8%) U = 11; D = 7	MDT‐CAM, CNS, Phy, Surg, Path, On	
e Virtual access saved travel time	Remote access has been helpful for some and has decreased travel time. Less travel, easier to access from home/other hospitals. (Physician)	11/252 (4%) U = 5; D = 6	CNS, Phy, Surg, MDT‐CAM, Onc	
f Virtual meetings solved venue issues	Creates space availability for other health care professionals to use our MDT room. (CNS)	4/252 (2%) U = 1; D = 3	CNS, MDT‐CAM, CNS, Surg	
g Virtual meetings reduced attendance (for some MDTs)	Reduced amount of people attending. (MDT‐CAM)	2/252 (1%) U = 0; D = 2	MDT‐CAM, Onc	
h Virtual meetings led to paperless documentation	Working remotely has allowed us to go fully paperless. (MDT‐CAM)	2/252 (1%) U = 1; D = 1	MDT‐CAM	
i Continuity of care	Continuity of Care. (Surgeon)	1/252 (0.4%) U = 1; D = 0	Surg	
j MDT guidelines produced	MDT guidelines produced to improve presentation and engagement. (Surgeon)	1/252 (0.4%) U = 1; D = 0	Surg	
k Re‐training	Yes, re‐training. (Oncologist)	1/252 (0.4%) U = 1; D = 0	Onc	
4. Changes that now seem more pressing		201/423 (48%) U = 137; D = 64		
None	None. (CNS)	83/201 (41%) U = 56; D = 26	MDT‐CAM, CNS, Phy, Surg, Onc,	
b Improving IT infrastructure, its use and access	Better IT support across sites. (AHP) Robust IT systems that are reliable and fit for purpose. (CNS)	43/201 (21%) U = 31; D = 12	Onc, Phy, Surg, CNS, MDT‐CAM, AHP, Rad	
c Streamlining of cases	Streamlining to create more time for complex cases. (MDT‐CAM)	32/201 (16%) U = 17; D = 15	Rad, Surg, MDT‐CAM, CNS, Onc, Phy, Path	
d Hybrid working	A hybrid model with the core members attending and multiple screens would be necessary. (Surgeon) Perhaps colleagues not in turn to attend the MDT meetings are able to dial into the MDT meeting virtually to present own cases. (Physician)	13/201 (7%) U = 8; D = 5	MDT‐CAM, Phy, Surg, CNS, Onc	
e More time for MDT meeting (due to increased workload) and a meeting break	Increase length of MDT—patient numbers have increased further and time for discussion for each patient is very limited. Need to introduce a short break in the MDT as with 50+ patients to discuss it is too long to concentrate without a break. (Oncologist)	10/201 (5%) U = 8; D = 2	Surg, CNS, MDT‐CAM, Phy, Onc	
f Improved preparation time	Need to get preparation of cases sorted so that it flows more smoothly. (Oncologist)	9/201 (5%) U = 6; D = 3	MDT‐CAM, Onc, Surg, CNS, Rad, Phy	
g Improved and better organised attendance and participation	Pathologists should not be attending. I could train a third grader to read my reports and answer 99% of questions they have. The other 1% could be answered by email. (Pathologist) Broader participation; committing to the time to participate. (Surgeon)	5/201 (3%) U = 5; D = 0	Path, Surg, Phy, Rad, MDT‐CAM	
h Improved referral process	Having cases discussed ASAP after referral, and appropriate feedback to referring team. (Surgeon)	2/201 (1%) U = 2; D = 0	MDT‐CAM, Surg	
i Recognition of MDT coordinator role	Larger role for MDT coordinator / pathway coordinator. (Surgeon)	2/201 (1%) U = 1; D = 1	Surg	
j Stricter guidelines around the number of patients for MDT meeting	Stricter guidelines around the amount of patients to be discussed and deadlines. (MDT‐CAM)	1/201 (0.5%) U = 1; D = 0	MDT‐CAM	
k Improving communication	Increase in working cross site has highlighted increased importance of communication. (AHP)	1/201 (0.5%) U = 1; D = 0	AHP	

*Note*: ^a^% of the total responses (*N* = 423). ^b^U = Number of responses from more local MDTs hosted largely at University and Teaching Hospitals; D = Number of responses from more specialist MDTs hosted largely at District Hospitals.

Abbreviations: AHP, Allied Health Professional; CNS, Cancer Nurse Specialist; MDT, Multi‐Disciplinary cancer Team; MDT‐CAM, Coordinators, Administrative support, and Management; Phy, Physician; Rad, Radiologist; Path, Pathologist; Surg, Surgeon.

### 3.3 Objective 2: Group comparisons of structured survey responses

**FIGURE 1:**
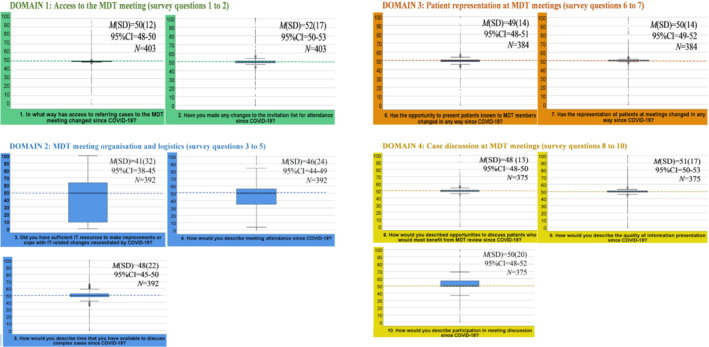
Boxplots representing distribution of answers across the survey questions

Figure 1 shows boxplots with the median survey responses and interquartile ranges (IQR) for the 10 questions (scored on the 0–100 rating scale) grouped into four (colour‐coded) domains.

Staff responses were relatively less variable (smaller IQR) on some questions, indicative of more consensus on the perception of ‘no change from before COVID‐19’ (median rating = 50). Specifically, staff consensus was clearer on *access to MDT meeting* (domain 1; q1 and 2), *patient representation at MDT meetings* (domain 3; q6 and 7), and *case discussion at MDT meetings* (domain 4; q8 and 9). On the other hand, despite a general perception of no change, staff responses were more variable (larger IQR) on *MDT meeting organisation and logistics* (domain 2; q3 and 4), and *case discussion at MDT meetings* (domain 4; q10). Amidst less consensus around the general perception of no change, we observed a wider range of staff ratings indicative of perceived improvement in *MDT meeting organisation and logistics* (domain 2; q3 and 4) since before COVID‐19. In contrast, we observed a wider range of staff ratings indicative of perceived worsening in *case discussion at MDT meetings* (domain 4; q10) from before COVID‐19 (Figure 1).

In line with Figure 1, *t*‐tests, performed on the composite domain scores, showed significantly lower scores, that is, improvement from before COVID‐19, for *MDT meeting organisation and logistics* (domain 2*; M* = 45, SD = 20) compared to the other three domains, that is, *access to MDT meetings* (domain 1; *M* = 50, SD = 12, *t*(390) = 5.028, *p* = 0.001), *case discussions at MDT meetings* (domain 4; *M* = 50, SD = 14, *t*(373) = −5.104, *p* = 0.001), and *patient participation* (domain 3; *M* = 50, SD = 12, *t*(382) = −4.537, *p* = 0.001), which showed no change from before COVID‐19 (i.e. all *M*s = 50, which is the mid‐point). Breakdown of descriptive statistics for the composite domain scores including the individual survey items can be found in Table 2.

**TABLE 2:** Descriptive statistics across composite scores and individual variables for the quantitative responses to the survey

	*N*	*M*	SD	95% CI for *M*		Mdn	IQR
				Lower	Upper		
DOMAIN 1: Access to the MDT meeting—Composite Score	403	50.36	11.87	49.16	51.57	50.00	1
1. In what way has access to referring cases to the MDT meeting changed since COVID‐19?		49.19	12.02	47.97	50.41	50.00	1
2. Have you made any changes to the invitation list for attendance since COVID‐19?		51.53	17.18	49.79	53.28	50.00	2
DOMAIN 2: MDT meeting organisation and logistics—Composite Score	**392**	**45.20**	**19.44**	**43.22**	**47.17**	**48.33**	**24**
3. Did you have sufficient IT resources to make improvements or cope with IT‐related changes necessitated by COVID‐19?		41.41	31.45	38.22	44.61	49.00	54
4. How would you describe meeting attendance since COVID‐19?		46.41	24.04	43.97	48.85	50.00	22
5. How would you describe time that you have available to discuss complex cases since COVID‐19?		47.66	22.28	45.50	49.93	50.00	5
DOMAIN 3: Patient representation—Composite Score	384	49.63	11.91	48.42	50.84	50.00	1
6. Has the opportunity to present patients known to MDT members changed in any way since COVID‐19?		49.09	13.63	47.70	50.47	50.00	2
7. Has representation of patients at meetings changed in any way since COVID‐19?		50.18	13.45	48.82	51.55	50.00	1
DOMAIN 4: Case discussion at MDT meetings—Composite Score	375	49.72	14.08	48.29	51.15	50.00	6
8. How would you described opportunities to discuss patients who would most benefit from MDT review since COVID‐19?		47.98	13.2	46.64	49.32	50.00	2
9. How would you describe quality of information presentation since COVID‐19?		51.15	16.72	49.45	52.85	50.00	2
10. How would you describe participation in meeting discussion since COVID‐19?		50.02	19.67	48.02	52.02	50.00	8

*Note*: In bold are significant differences.

Abbreviations: CI, Confidence Interval; IQR, Interquartile Range; *M*, Mean; Mdn, Median; *N*, Number; SD, Standard Deviation.

## 4 DISCUSSION

The aim of the study was to explore quantitatively and qualitatively the views of MDT members on the impact of COVID‐19 on their meetings with focus on (1) access to MDT meetings, (2) meeting organisation and logistics, (3) patient representation at the meetings, and (4) quality of case reviews.

While overall there was no prominent evidence of perceived worsening from before COVID‐19 in any of the domains investigated by the survey, the boxplots do offer some indication in the direction of perceived worsening in the ability to participate in MDT meeting discussion since COVID‐19 (see Figure 1, Question 10). This has also been mentioned in the qualitative responses with 12% of the MDT members reporting challenges around team communication and case discussion due to the virtual or hybrid nature of the meetings.

Moreover, we found that the perception of MDT meeting organisation and logistics significantly improved since COVID‐19. Indeed, hybrid working and virtual attendance was the most frequent theme emerging in terms of changes that will be maintained going forward and the changes since COVID‐19 that provided solutions—84% of respondents agreed with this change. Improved IT infrastructure and streamlining were also one of the top perceived improvements in terms of meeting organisation and logistics. For example, the electronic referrals and paperless documentation were one of the improvements that made MDT working more efficient as a result of COVID‐19‐necessitated changes. Consistently across professional groups it was also clear that the changes that provided solutions were around streamlining of meeting discussion and cases alongside the improvements in IT infrastructure, allowing more time for complex cases and more focused discussions. Our data suggested that despite the initial challenges with the IT that impacted duration of meetings and caseload and caused pressure to cancer teams, the subsequent substantial improvements in the IT infrastructure, its access and use were indeed made because of COVID‐19 and the need to facilitate remote working.

In terms of the wider literature on MDT functioning, a survey study of UK colorectal cancer teams in 2007 found that 30% had telemedicine facilities available, but only 17/32 of these teams used such facilities for remote MDT work.^28^ Since then, this technology has been increasingly used in MDT meetings in the UK and in other countries.^29^, ^30^ Our results suggest that since the COVID‐19 pandemic, the use of videoconferencing in MDT meetings among respondents is now universal. Authors have commented that given the restrictions on personal interaction, video‐conferenced MDT meetings provide a good alternative to face‐to‐face meetings.^11^, ^12^ The implications of such improved access to videoconferencing needs to be appraised considering wider evidence regarding how effective video‐conferenced cancer MDTs are, which has been studied prior to the emergence of the pandemic.

Moreover, a systematic review by Lamb et al in 2011^31^ explored the effect of telemedicine on care management decisions in MDT meetings. The authors found that telemedicine can improve meeting attendance, and it does not appear to negatively affect care. More recently, Horlait et al^29^ also found that teleconferencing or videoconferencing can improve meeting attendance rates. The study also found that remote interaction facilitated communication between team members who are physically distant from each other.^29^ This was similar to findings by Jannsen and colleagues^30^ who listed advantages of video and teleconferencing technology included the ability to include teams with dispersed members, valuable discussions with specialist colleagues, the potential reduced treatment time for patients and improved coordination of care, and improved access of primary care providers and patients with specialist care.^30^ Other important but less tangible benefits were postulated to arise from developing a shared best practice and peer‐review among specialists in different local teams within a spread out geographical area, brought together remotely when they would otherwise not get a chance to interact.^32^ Virtual MDTs also chyme with the ‘greener NHS’ agenda—a drive towards a net zero national health service—and present a potential benefit with going paperless and reducing travel, potentially reducing energy utilisation.^33^

Videoconferencing also has disadvantages. In previous studies, these have included concerns about privacy and confidentiality of patient data, the need for additional preparation for MDT meetings, challenges in coordinating meeting times and unreliability of technology,^29^, ^30^ which with the above findings, are in keeping with the results of our own study. Use of a checklist specifically for video‐conferenced MDT meetings has been proposed as a means of addressing such issues.^11^ Whether video‐conferenced MDT meetings are cost effective appears to be unclear. The infrastructure costs to allow remote MDT working can be significant but has been suggested to become cost‐neutral once utilisation reaches 20–30 meetings per year.^34^ Jannsen et al.^30^ found that there was also evidence of cost concerns from the implementation and adoption of virtual MDTs, whereas Horlait et al.^29^ concluded that virtual MDTs can save money. Interestingly, Alexanderson et al.^32^ assessed use of video equipment and found that it increased the costs (273 EUR/case for video‐based meetings vs. 169 EUR/case for local meetings). They commented that higher costs for video‐conferenced MDTs might be offset by reduced travel time for members and improvements in care quality due to the treating physicians being able to attend MDTs.^32^

The results of the current study suggest that MDT members perceive that the increased use of teleconferencing and remote MDT access in response to the COVID‐19 pandemic has not adversely affected access to the meeting, case discussions, or patient representation. They also suggest that MDT meeting organisation and logistics significantly improved since COVID‐19, and that MDTs had sufficient IT resources to make improvements and cope with the IT‐related changes. This was reflected in the qualitative findings where it was evident that despite the initial challenges with the IT that impacted duration of meetings and caseload and have caused stress, the subsequent substantial improvements in the IT infrastructure, its access and use were indeed made because of COVID‐19 and the need to facilitate remote working.

### 4.1 Implications and generalizability

Our findings are important to consider in the current climate with multiple pressures on healthcare teams, such as financial pressures,^14^, ^15^ to staff shortages,^17^ increasing cancer incidence,^14^, ^16^ growing workload,^18^ and human factors.^19^, ^20^, ^21^, ^22^, ^23^, ^24^ Given the large estimated cost of MDTs (over £100 million per year in the UK) understanding what impacts its performance and how it can be made safer and more efficient is critical to continuous quality improvement efforts.^35^, ^36^ Indeed, just prior to the pandemic, NHS England completed a pilot study of the effect of streamlining MDT meetings and published national guidance regarding the implementation of streamlining in MDT meetings in order to enable cancer MDTs in England to adapt to increasing demand. This guidance encourages MDTs to be selective about which cases are discussed in full at the MDT meeting, favouring complex cases with simpler cases being treated according to an agreed standard of care. The results of the present study suggest that the ability to discuss cases that benefit most from MDT review, that is, complex patients, might have improved with the changes to remote access and online meetings. Importantly, our qualitative findings point to an increasing volume and complexity of oncology patients that need to be addressed further supporting a more streamlined approach. The present study also suggests that IT infrastructure will be critical to support these changes and facilitate further improvement with innovations such as streamlining.

Taken together, these findings suggest the changes that have been necessary to continue service during the COVID‐19 pandemic might have the unintended but desirable effect of facilitating a streamlined agenda. Failure of healthcare organisations to provide the necessary IT infrastructure could compromise the ability of MDTs to function normally during the COVID‐19 period, let alone to make the necessary steps to successfully improve the effectiveness and efficiency of MDT working. In particular, the MDTs went through several agile reorganisations during COVID‐19 to keep diagnostic and treatment pathways functioning. As a result of this, the governance function of MDTs may have taken on more importance to ensure excellent patient oversight and tracking was still maintained when other healthcare processes were more fluid, and resources diverted towards COVID‐19. This requirement for a higher level of governance may have made MDTs less likely to introduce other changes the teams themselves wanted to prioritise, given the unusual circumstances of a world pandemic raging around them. But going forward, teams need to be supported to address areas important to them such as streamlining. There are various teamworking tools^36^, ^37^ available to support this change but the involvement of hospital management is paramount early on, to facilitate this.

Replication and assessment of generalizability of the findings to other cancers and health systems needs to be examined to determine the extent to which they apply to them. Indeed, other national healthcare systems such as the Veterans Health Administration in the United States, have explored the impact of COVID‐19 on virtual oncology care, including tumour boards, finding both opportunities and challenges corroborating our findings to some extent.^38^ Similarly, Sniderman et al utilised mixed methods to describe impact on paediatric cancer providers during the pandemic, capturing some themes that overlap with our findings; most notably around staffing challenges.^39^

### 4.2 Limitations

The results of the study should be interpreted in light of potential limitations. First, this study relies on self‐report obtained via a snowball sampling approach with most responses coming from England, UK, which may reduce representativeness of findings and reduce wider generalizability. Snowball sampling is a non‐probability sampling method where new participants are recruited by other participants to form part of the sample. Advantages of this method include low‐cost and ability to target potential participants unknown to the researchers through existing networks. The number of responses exceeded that which the research team would have been able to achieve through direct recruitment, thus increasing the sample and representation of the population. However, limitations must be acknowledged: as the sample is not chosen through random selection of the population being studied, it may not be entirely representative. Moreover, researchers have little or no control over the sampling process (other than the instructions in the brief sent to potential participants) and rely mainly on referrals from already‐identified participants. Since people refer others whom they know (and share traits with), snowballing can have a potential selection bias.

Second, the total number of participants is certainly only a fraction of the thousands of healthcare professionals working in the thousands of MDTs in the United Kingdom, and may favour those with a tendency to respond to such a survey (e.g. those with strong opinions). Third, the availability of demographic data on participants is limited. Forth, it should be acknowledged that our subgroup quantitative analyses were not undertaken according to whether the respondents were from district hospital and university/teaching hospitals—this is because we did not find statistical significance between the two groups (as reported in the Results/Participant Characteristics above). We cannot, therefore, comment on any possible quantitative differences in the results according to the situation of respondents. Lastly, the study aimed to explore perceptions of changes caused by the pandemic relating specifically to the MDT meetings, and not to the comprehensive working of MDTs in patient care as whole. Although MDT meetings represent one important segment of the care pathway, there are many other areas of MDT working that could have been impacted by COVID‐19. These limitations, when taken together, could limit the generalizability of the findings.

### 4.3 Further research

Further research is needed to validate current MDT quality improvement tools and approaches^36^, ^38^ on the virtual/hybrid MDT meetings to enable continuous measurement and improvement going forward to maximise value for all stakeholders, including patients. For example, the indication from the current study that the ability to participate in the meeting case discussion may have gotten worse since COVID‐19 and the introduction of virtual/hybrid MDT meetings should be followed up using, for example, observational methodology and currently available tools for assessing the focus and quality of MDT patient reviews.^27^ In particular, given that virtual MDT meetings can improve attendance,^29^, ^30^ further research should explore, using the current tools, the quality of contribution to discussion between face‐to‐face and virtual (or hybrid) meetings. Future research should also further explore the quality of decision‐making and communication in MDT meetings pre and post COVID‐19 pandemic. This could be achieved using a retrospective‐prospective approach with the post‐pandemic data collected prospectively and benchmarking it against the existing literature, or to the pre‐pandemic MDT datasets available of data repositories (e.g. decision‐making, interactions and complexity across three cancer multidisciplinary teams, Zenodo).^40^ Lastly, the impact of COVID‐19 may have been different according to the situation of the Hospital, and whether they were a specialist‐MDT, at the hub, or a local‐MDT, at the spoke end of the organisation—although we did not find statistical significance between the two groups, further research could nonetheless investigate this hypothesis in more detail.

## 5 CONCLUSION

This study explored the perception of change since COVID‐19 among cancer MDT members using a mixed methods approach. While overall we found no significant perceived worsening across the four domains investigated—access to the MDT meeting, meeting organisation and logistics, patient representation, and case discussion—there was an indication in this direction for the latter warranting further ‘live’ assessments of MDT meetings. We found significant perceived improvements in the meeting organisation and logistics. Further studies to replicate these results in cancer services outside the UK and of how newly established virtual MDTs deliver care compared to face‐to‐face ones are warranted.

## AUTHOR CONTRIBUTIONS

**Tayana Soukup:** Conceptualization (equal); data curation (equal); formal analysis (equal); methodology (equal); project administration (equal); validation (equal); writing – original draft (equal); writing – review and editing (equal). **David Winters:** Conceptualization (equal); investigation (equal); methodology (equal); project administration (equal); writing – original draft (equal); writing – review and editing (equal). **Kia‐Chong Chua:** Formal analysis (equal); methodology (equal); supervision (equal); writing – original draft (equal); writing – review and editing (equal). **Philip Rolland:** Data curation (equal); project administration (equal); writing – original draft (equal); writing – review and editing (equal). **Jacqueline Moneke:** Conceptualization (equal); project administration (equal); writing – original draft (equal); writing – review and editing (equal). **Ted Skolarus:** Investigation (equal); methodology (equal); project administration (equal); resources (equal); writing – original draft (equal); writing – review and editing (equal). **Rasiah Bharathan:** Conceptualization (equal); investigation (equal); project administration (equal); writing – original draft (equal); writing – review and editing (equal). **Leanne Harling:** Investigation (equal); project administration (equal); writing – original draft (equal); writing – review and editing (equal). **Anish Bali:** Investigation (equal); project administration (equal); resources (equal); writing – original draft (equal); writing – review and editing (equal). **Viren Asher:** Investigation (equal); project administration (equal); resources (equal); writing – original draft (equal); writing – review and editing (equal). **Tasha Gandamihardja:** Investigation (equal); project administration (equal); resources (equal); writing – original draft (equal); writing – review and editing (equal). **Nick Sevdalis:** Investigation (equal); methodology (equal); writing – original draft (equal); writing – review and editing (equal). **James Green:** Conceptualization (equal); investigation (equal); methodology (equal); project administration (equal); resources (equal); supervision (equal); writing – original draft (equal); writing – review and editing (equal). **Benjamin Lamb:** Conceptualization (equal); data curation (equal); formal analysis (equal); investigation (equal); methodology (equal); project administration (equal); resources (equal); supervision (equal); validation (equal); writing – original draft (equal); writing – review and editing (equal).

## FUNDING INFORMATION

TS research is supported by the Welcome Trust (219425/Z/19/Z) and Diabetes UK (reference 19/0006055). NS research is funded by the NIHR via the ‘Applied Research Collaboration: South London’ at King's College Hospital NHS Foundation Trust, London, UK. NS is also a member of King's Improvement Science, which offers co‐funding to the NIHR ARC South London and is funded by King's Health Partners (Guy's and St Thomas' NHS Foundation Trust, King's College Hospital NHS Foundation Trust, King's College London and South London and Maudsley NHS Foundation Trust), and the Guy's and St Thomas' Foundation. The funding agreement ensured the authors' independence in designing the study, interpreting the data, writing, and publishing the report. The views expressed are those of the authors and not necessarily those of the NHS, NIHR, charity or the Department of Health and Social Care.

## CONFLICT OF INTEREST

BL and TS received funding from Cancer Alliance and Health Education England for training MDTs in assessment and quality improvement methods in the United Kingdom. TS received consultancy fees from Roche Diagnostics. NS is the Director of London Safety & Training Solutions Ltd, which offers training in patient safety, implementation solutions and human factors to healthcare organisations and the pharmaceutical industry. JG is the Director of Green Cross Medical Ltd that developed MDT FIT for use by National Health Service Cancer Teams in the UK. The other authors have no conflicts of interest to report.

## ETHICS APPROVAL AND CONSENT TO PARTICIPATE

The study was registered as service evaluation or audit at each site. Consent was implied upon return of the survey, and response to the survey was voluntary, in accordance with the Good Clinical Practice and Clinical Governance.

## Supporting information


File S1

File S2

## Data Availability

The anonymised dataset supporting this study is available in Zenodo, a research data repository, https://doi.org/10.5281/zenodo.6369996, under the Creative Commons Attribution Non‐Commercial Non‐Derivative 4.0 licence. Researchers are free to reuse and redistribute the data set on the condition that they attribute it, that they do not use it for commercial purposes, and that they do not alter it. For any reuse or redistribution, researchers must make clear to others the licence terms of this work and reference the dataset accordingly.
