# Correction to “Evaluation of changes to work patterns in multidisciplinary cancer team meetings due to the COVID‐19 pandemic: A national mixed‐method survey study”

**DOI:** 10.1002/cam4.6529

**Published:** 2023-09-27

Soukup T, Winters D, Chua K‐C, et al. Evaluation of changes to work patterns in multidisciplinary cancer team meetings due to the COVID‐19 pandemic: A national mixed‐ method survey study. Cancer Med. 2023;12:8729–8741. doi:10.1002/cam4.5608

In the originally published article, the fourth author's name had been misspelled as “Philip Rowland.” It is now corrected to “Philip Rolland.”

The updated author byline and affiliations are mentioned below:

**Tayana Soukup^1^** | **David Winters^2^** | **Kia‐Chong Chua^1^** | **Philip Rolland^3,4^** | **Jacqueline Moneke^3^** | **Ted A. Skolarus^5^** | **Rasiah Bharathan^6^** | **Leanne Harling^7,8^** | **Anish Bali^9^** | **Viren Asher^9^** | **Tasha Gandamihardja^10^** | **Nick Sevdalis^1^** | **James S. A. Green^2^** | **Benjamin W. Lamb^2,11,12^**

^1^Institute of Psychiatry, Psychology, and Neuroscience, Health Service and Population Research Department, King's College London, London, UK

^2^Department of Urology, Barts Health, NHS Trust, London, UK

^3^Department of Urology, Cambridge, University Hospital NHS Trust, London, UK

^4^Gloucestershire Hospitals NHS Foundation Trust, Cheltenham, UK

^5^Dow Division of Health Services, Research, Department of Urology, University of Michigan, Center for Clinical Management Research, Veterans Affairs Ann Arbor Healthcare System, Ann Arbor, Michigan, USA

^6^University Hospitals of Leicester NHS, Trust, Leicester, UK

^7^Department of Surgery and Cancer, Imperial College London, London, UK

^8^School of Cancer and Pharmaceutical Science, Kings College London, London, UK

^9^Gynaecology Cancer Centre, University Hospitals of Derby & Burton, Derby, UK

^10^Chelmsford Breast Unit, Broomfield Hospital, Chelmsford, UK

^11^Bart's Cancer Institute, Queen Mary, University of London, London, UK

^12^Department of Urology, University London College Hospitals, London, UK

We apologize for this error.

