# Supplementary material for: Evaluation of changes to work patterns in multidisciplinary cancer team meetings due to the COVID‐19 pandemic: A national mixed‐method survey study
Source: Cancer Med. 2023 Jan 17;12(7):8729–41. doi: 10.1002/cam4.5608 (PMC10134365; doi:10.1002/cam4.5608)
Supplement: Supplementary file 1 — File S1 [file CAM4-12-8729-s002.docx]

## ADDITIONAL FILE 1: INVITATION EMAIL

Dear Colleagues,

I would be grateful if you would participate in a very brief survey to help gauge impact of COVID-19 on cancer MDTs. Your participation will help broaden our understanding of the impact of COVID-19 on MDT functioning, and on Urological cancer MDTs specifically.

This survey will not collect any person identifiable information. Your answers will be kept entirely anonymous, and the data will be used to gain better understand of MDT functioning post-Covid. Published report will be entirely anonymous.

The survey will take approximately 5 minutes of your time. When filling in the survey please think about your MDTs and respond to the questions presented in this survey. There are no right or wrong answers. Please try to be as honest as possible.

***We would be very grateful if you could forward this email, along with the survey link to other members of your MDT. In particular we want to hear the views of MDT coordinators, who are integral to the functioning of MDTs, but can be difficult to recruit.***

Your participation is entirely voluntary. By completing this survey, you are consenting to participate in this study.

<https://www.surveymonkey.co.uk/r/KCX2NMG>

Thank you very much for your help and taking the time to complete this survey.

**[NAME OF COLABORATOR]**
