# Supplementary material for: Evaluation of changes to work patterns in multidisciplinary cancer team meetings due to the COVID‐19 pandemic: A national mixed‐method survey study
Source: Cancer Med. 2023 Jan 17;12(7):8729–41. doi: 10.1002/cam4.5608 (PMC10134365; doi:10.1002/cam4.5608)
Supplement: Supplementary file 2 — File S2 [file CAM4-12-8729-s001.docx]

## ADDITIONAL FILE 2: COPY OF THE SURVEY

**About you**

- - Your main cancer speciality/interest (please specify)
  - Your current position within the organisation (please specify)
  - Years in the current position (please specify)
  - Type of Organization you work in (University or Teaching Hospital / District Hospital /other please specify)
  - Name of City or Town (please specify)
  - Country (please specify)

**Qualitative open questions**

1. What changes to MDT working will you maintain? (Free text)
2. Have changes made to MDT working since COVID-19 caused any specific problems? (Free text**)**
3. Have changes made to MDT working since COVID-19 provided any solutions? (Free text)
4. Are there any other changes to make to MDT working that now seem more pressing? (Free text)

**DOMAIN 1: Access to the MDT meeting**

1. In what way has access to referring cases to the MDT meeting changed since COVID-19?
   - - - improved – no different- worsened [0-100 sliding scale marker]
2. Have you made any changes to the invitation list for attendance since COVID-19?
   - - - widened – no difference – reduced [0-100 sliding scale marker]

**DOMAIN 2: MDT Meeting organisation and logistics**

1. Did you have sufficient IT resources to make improvements or cope with IT-related changes necessitated by COVID-19?
   - - - Yes- somewhat- no [0-100 sliding scale marker]
2. How would you describe meeting attendance since COVID-19?
   - - - improved – no change- worsened [0-100 sliding scale marker]
3. How would you describe time that you have available to discuss complex cases since COVID-19?
   - - - Sufficient- no change- insufficient [0-100 sliding scale marker]

**DOMAIN 3: Patient representation at MDT meetings**

1. Has the opportunity to present patients known to MDT members changed in any way since COVID-19?
   - - - Improved- no change- declined [0-100 sliding scale marker]
2. Has the representation of patients at meetings changed in any way since COVID-19?
   - - - Improved- no change- declined [0-100 sliding scale marker]

**DOMAIN 4: Case discussion at MDT meetings**

1. How would you described opportunities to discuss patients who would most benefit from MDT review since COVID-19?
   - - - Improved- no change- declined [0-100 sliding scale marker]
2. How would you describe the quality of information presentation since COVID-19?
   - - - Improved- no change- declined [0-100 sliding scale marker]
3. How would you describe participation in meeting discussion since COVID-19?
   - - - widened – no difference – reduced [0-100 sliding scale marker]
